# Supplementary material for: Place susceptibility index mapping at local government scale from population-based survey for Sub-Saharan Africa
Source: PLoS One. 2026 Feb 17;21(2):e0340782. doi: 10.1371/journal.pone.0340782 (PMC12912589; doi:10.1371/journal.pone.0340782)
Supplement: S1 Tables — (DOCX) [file pone.0340782.s001.docx]

# Supplemental material

## Supplemental Table 1. Geospatial Covariates Used in SVI Computation

| **Geocovariates** | **Name** | **Source** | **Year** |
| --- | --- | --- | --- |
| NLIGHTS | Nighttime Lights Intensity | Wordpop.org | 2016 |
| LST | Land Surface Temperature | Google Earth Engine | *Year of the survey |
| SLOPE | Terrain Slope | Wordpop.org | 2015 |
| EVI | Enhanced Vegetation Index | Google Earth Engine | *Year of Survey |
| ACCESS | Accessibility Index | Wordpop.org | 2016 |
| DMROADS | Distance to Major Roads | Wordpop.org | 2016 |
| DMROADSINT | Distance to Road Intersections | Wordpop.org | 2016 |
| POPPP | Population Per Pixel | Wordpop.org | *Year of Survey |
| NDVI | Normalized Difference Vegetation Index | Google Earth Engine | *Year of Survey |
| DMWATER | Distance to Water Bodies | Wordpop.org | 2016 |
| ELEV | Elevation | Wordpop.org | 2015 |

NB. All Geocovariates were computed at 100m spatial resolution. *For each country the geocovariates for the year of the survey were extracted from monthly or annual aggregate data.

## Supplemental Table 2. Computed Variables and their descriptions

| **Computed Variable** | **Description** |
| --- | --- |
| Btwatersource | Better household water source |
| Bttoilettype | Noecosrept |
| Ytoiletshare | Shared toilet facility |
| Yhaveelect | Household has electricity |
| yhaveradio | Household owns a working radio |
| yhavetele | Household owns a working television |
| yhavecell | Household owns a working mobile phone |
| yhaverefrig | Household owns a working refrigerator |
| btcookingfuel | Better cooking fuel type |
| btmatfloor | Better floor material |
| btmatroof | Better roof material |
| btmatexwalls | Better exterior wall material |
| yownmoto | Household owns a working motorcycle/scooter |
| Yowncar | Household owns a working car |
| yownknapep_ng | Households own a working keke napep (tricycle) |
| noecosrept | Household received economic support |
| womenprop | Proportion of women in the population |
| rel_literacy | Higher educational attainment |
| employed_12mth | Employed in the past 12 months |
| employed_7days | Employed in the past 7 days |
| mar_age | Age at first marriage |
| mar_witpartner | Currently married or living with a partner |
| prghlthaccess | Visited health facility for antenatal care |
| prgnow_prop | Proportion of women currently pregnant |
| prp3msick | Sick for at least 3 months in the past year |
| prpelderly | Proportion of elderly individuals in the population |
| prpyoung | Proportion of young individuals (0-14 years) in the population |

## Supplemental Table 3. Indicators for Zambia

| **Variable Computed** | **Variable Used** | **Extracted From** | **Name** | **Code** | **Computation** |
| --- | --- | --- | --- | --- | --- |
| btwatersource | watersource | Household Interview | source of water | 11= piped into dwelling | Proportion |
|  |  |  |  | 12 = piped into yard/plot |  |
|  |  |  |  | 13 = public tap/standpipe |  |
|  |  |  |  | 21 = tube well or borehole |  |
|  |  |  |  | 31 = protected well |  |
|  |  |  |  | 41 = protected spring |  |
|  |  |  |  | 91 = bottled water |  |
| bttoilettype | toilettype |  | type of toilet facility | 11 = flush or pour flush toilet | Proportion |
|  |  |  |  | 22 = ventilated improved pit latrine |  |
| ytoiletshare | toiletshare |  | shared toilet facility | 1 = yes | Proportion |
| yhaveelect | hhqitems_a |  | availability of electricity | 1 = yes | Proportion |
| yhaveradio | hhqitems_b |  | available working radio | 1 = yes | Proportion |
| yhavetele | hhqitems_c |  | available working television | 1 = yes | Proportion |
| yhavecell | hhqitems_d |  | available working mobile phone | 1 = yes | Proportion |
| yhaverefrig | hhqitems_e |  | available working refrigerator | 1 = yes | Proportion |
| btcookingfuel | cookingfuel |  | Type of cooking fuel | 1 = electricity | Proportion |
|  |  |  |  | 2 = lpg / natural gas |  |
| btmatfloor | matfloor |  | material of floor | 33 = ceramic tiles | Proportion |
|  |  |  |  | 34 = cement |  |
|  |  |  |  | 35 = carpet |  |
| btmatroof | matroof |  | material of roof | 21 = corrugated iron | Proportion |
|  |  |  |  | 31 = asbestos sheet / cement fiber |  |
|  |  |  |  | 32 = concrete |  |
| btmatexwalls | matexwalls |  | material of exterior wall | 31 = cement | Proportion |
|  |  |  |  | 32 = stone with lime/ cement |  |
|  |  |  |  | 33 = bricks |  |
|  |  |  |  | 34 = cement blocks |  |
| yownmoto | hhqown_b |  | available working motor cycle / scooter | 1 = yes | Proportion |
| yowncar | hhqown_c |  | available working car | 1 = yes | Proportion |
| noecosrept | econsup12_a |  | economic support within 12 months | 1 = yes | Proportion |
| womenprop | gender | Adult Individual Interview | proportion of women | 2 = gender | Proportion |
| rel_literacy | schcom |  | highest level of school completed | 2 = lvl _1 (primary, 1-7) | Proportion |
|  |  |  |  | 3 = lvl_2 (secondary, 1-3 or A-B) |  |
|  |  |  |  | 4 = lvl_2 (high school, 4-5 or D-E) |  |
|  |  |  |  | 5 = lvl_3 (short cycle tertiary) |  |
|  |  |  |  | 6 = lvl_3 (bachelor's program) |  |
|  |  |  |  | 7 = lvl_3 (master's program) |  |
|  |  |  |  | 8 = lvl_3 (doctoral program) |  |
| employed_12mth | work12mo |  | work done in the last 12 months | 1 = yes | Proportion |
| employed_7days | work7days |  | work done in the last 7 days | 1 = yes | Proportion |
| mar_age | agemar |  | age when first marriage occurred | > 0 | Median |
| mar_witpartner | curmar |  | marital status | 1 = married | Proportion |
|  |  |  |  | 2 = living together |  |
| prghlthaccess | prgcare |  | health facility visit for antenatal care | 1 = yes | Proportion |
| prgnow_prop | pregnant |  | pregnancy status | 1 = yes | Proportion |
| prp3msick | sick3mo | Household Roster | Has been very sick for at least 3 months during the past 12 months | 1 = Yes | Proportion |
| prpelderly | agegroup5population |  | proportion of elderly | integer | Proportion |
| prpyoung | agegroup5population |  | proportion of young (0 to 14 years) | integer | Proportion |

## Supplemental Table 4. Indicators for Mozambique

| **Variable Computed** | **Variable Used** | **Extracted From** | **Name** | **Code** | **Computation** |
| --- | --- | --- | --- | --- | --- |
| btwatersource | watersource | Household Interview | source of water | 11= piped into dwelling | Proportion |
|  |  |  |  | 12 = piped into yard/plot |  |
|  |  |  |  | 13 = public tap/standpipe |  |
|  |  |  |  | 21 = tube well or borehole |  |
|  |  |  |  | 31 = protected well |  |
|  |  |  |  | 41 = protected spring |  |
|  |  |  |  | 91 = bottled water |  |
| bttoilettype | toilettype_mz |  | type of toilet facility | 11 = flush or pour flush toilet | Proportion |
|  |  |  |  | 21 = traditional pit laterine |  |
|  |  |  |  | 22 = ventilated improved pit laterine |  |
|  |  |  |  | 23 = improved latrine |  |
| ytoiletshare | toiletshare |  | shared toilet facility | 1 = yes | Proportion |
| yhaveelect | hhqitems_a |  | availability of electricity | 1 = yes | Proportion |
| yhaveradio | hhqitems_b |  | available working radio | 1 = yes | Proportion |
| yhavetele | hhqitems_c |  | available working television | 1 = yes | Proportion |
| yhavecell | hhqitems_d |  | available working mobile phone | 1 = yes | Proportion |
| yhaverefrig | hhqitems_e |  | available working refrigerator | 1 = yes | Proportion |
| btcookingfuel | cookingfuel |  | Type of cooking fuel | 1 = electricity | Proportion |
|  |  |  |  | 2 = lpg / natural gas |  |
|  |  |  |  | 3 = biogas |  |
|  |  |  |  | 4 = paraffin / kerosene |  |
| btmatfloor | matfloor |  | material of floor | 31 = parquet or polished wood | Proportion |
|  |  |  |  | 32 = vinyl tiles / vinyl carpet |  |
|  |  |  |  | 33 = ceramic tiles |  |
|  |  |  |  | 34 = cement |  |
|  |  |  |  | 35 = carpet |  |
| btmatroof | matroof |  | material of roof | 21 = corrugated iron | Proportion |
|  |  |  |  | 22 = tin cans |  |
|  |  |  |  | 31 = asbestos sheet / cement fiber |  |
|  |  |  |  | 32 = concrete |  |
|  |  |  |  | 33 = ceramic / clay tiles |  |
| btmatexwalls | matexwalls |  | material of exterior wall | 31 = cement | Proportion |
|  |  |  |  | 32 = stone with lime/ cement |  |
|  |  |  |  | 33 = bricks |  |
|  |  |  |  | 34 = cement blocks |  |
| roomsleepHH | roomsleep, rostercount |  | number of rooms for sleeping | integer | Median |
| yownmoto | hhqown_b |  | available working motor cycle / scooter | 1 = yes | Proportion |
| yowncar | hhqown_c |  | available working car | 1 = yes | Proportion |
| noecosrept | econsup12_a |  | economic support within 12 months | 1 = yes | Proportion |
| childHHratio | childcount, rostercount |  | number of children per household | integer | Median |
| HHSize | rostercount |  | size of household | integer | Median |
| womenprop | gender | Adult Interview | proportion of women | 2 = gender | Proportion |
| rel_literacy | schcom_mz |  | highest level of school completed | 3 = lvl_2 (secondary, 1-3 or A-B) | Proportion |
|  |  |  |  | 4 = lvl_2 (high school, 4-5 or D-E) |  |
|  |  |  |  | 5 = lvl_3 (short cycle tertiary) |  |
|  |  |  |  | 6 = lvl_3 (bachelor's program) |  |
|  |  |  |  | 7 = lvl_3 (master's program) |  |
|  |  |  |  | 8 = lvl_3 (doctoral program) |  |
| employed_12mth | work12mo |  | work done in the last 12 months | 1 = yes | Proportion |
| employed_7days | work7days |  | work done in the last 7 days | 1 = yes | Proportion |
| mar_age | agemar |  | age when first marriage occurred | > 0 | Median |
| mar_witpartner | curmar |  | marital status | 1 = married | Proportion |
|  |  |  |  | 2 = living together |  |
| prghlthaccess | prgcare |  | health facility visit for antenatal care | 1 = yes | Proportion |
| prgnow_prop | pregnant |  | pregnancy status | 1 = yes | Proportion |
| prp3msick | sick3mo | Household Roster | Has been very sick for at least 3 months during the past 12 months | 1 = Yes | Proportion |
| prpelderly | agegroup5population |  | proportion of elderly | integer | Proportion |
| prpyoung | agegroup5population |  | proportion of young (0 to 14 years) | integer | Proportion |

## Supplemental Table 5. Indicators for Malawi

| **Variable Computed** | **Variable Used** | **Extracted From** | **Name** | **Code** | **Computation** |
| --- | --- | --- | --- | --- | --- |
| btwatersource | watersource | Household Interviews | source of water | 11= piped into dwelling | Proportion |
|  |  |  |  | 12 = piped into yard/plot |  |
|  |  |  |  | 13 = public tap/standpipe |  |
|  |  |  |  | 21 = tube well or borehole |  |
|  |  |  |  | 31 = protected well |  |
|  |  |  |  | 41 = protected spring |  |
|  |  |  |  | 91 = bottled water |  |
| bttoilettype | toilettype |  | type of toilet facility | 11 = flush or pour flush toilet | Proportion |
|  |  |  |  | 21 = traditional pit laterine |  |
|  |  |  |  | 22 = ventilated improved pit laterine |  |
| ytoiletshare | toiletshare |  | shared toilet facility | 1 = yes | Proportion |
| yhaveelect | hhqitems_a |  | availability of electricity | 1 = yes | Proportion |
| yhaveradio | hhqitems_b |  | available working radio | 1 = yes | Proportion |
| yhavetele | hhqitems_c |  | available working television | 1 = yes | Proportion |
| yhavecell | hhqitems_d |  | available working mobile phone | 1 = yes | Proportion |
| yhaverefrig | hhqitems_e |  | available working refrigerator | 1 = yes | Proportion |
| btcookingfuel | cookingfuel |  | Type of cooking fuel | 1 = electricity | Proportion |
|  |  |  |  | 2 = lpg / natural gas |  |
|  |  |  |  | 3 = biogas |  |
|  |  |  |  | 4 = paraffin / kerosene |  |
| btmatfloor | matfloor |  | material of floor | 31 = parquet or polished wood | Proportion |
|  |  |  |  | 32 = vinyl tiles / vinyl carpet |  |
|  |  |  |  | 33 = ceramic tiles |  |
|  |  |  |  | 34 = cement |  |
|  |  |  |  | 35 = carpet |  |
| emp | matroof |  | material of roof | 21 = corrugated iron | Proportion |
|  |  |  |  | 22 = tin cans |  |
|  |  |  |  | 31 = asbestos sheet / cement fiber |  |
|  |  |  |  | 32 = concrete |  |
|  |  |  |  | 33 = ceramic / clay tiles |  |
| btmatexwalls | matexwalls |  | material of exterior wall | 31 = cement | Proportion |
|  |  |  |  | 32 = stone with lime/ cement |  |
|  |  |  |  | 33 = bricks |  |
|  |  |  |  | 34 = cement blocks |  |
| roomsleepHH | roomsleep / rostercount |  | number of rooms for sleeping | integer | Median |
| yownmoto | hhqown_b |  | available working motor cycle / scooter | 1 = yes ( B = A working motor cycle / scoooter | Proportion |
| yowncar | hhqown_c |  | available working car | 1 = yes ( C = A working car or truck) | Proportion |
| noecosrept | econsup12_a |  | economic support within 12 months | 1 = yes ( A = nothing) | Proportion |
| childHHratio | childcount/rostercount |  | number of children per house hold | Integer | Median |
| HHSize | rostercount |  | size of household | integer | Median |
| womenprop | gender | Adult Inerviews | proportion of women | 2 = female | Proportion |
| rel_literacy | schcom |  | highest level of school completed | 2 = lvl _1 (primary, 1-7) | Proportion |
|  |  |  |  | 3 = lvl_2 (secondary, 1-3 or A-B) |  |
|  |  |  |  | 4 = lvl_2 (high school, 4-5 or D-E) |  |
|  |  |  |  | 5 = lvl_3 (short cycle tertiary) |  |
|  |  |  |  | 6 = lvl_3 (bachelor's program) |  |
|  |  |  |  | 7 = lvl_3 (master's program) |  |
|  |  |  |  | 8 = lvl_3 (doctoral program) |  |
| employed_12mth | work12mo |  | work done in the last 12 months | 1 = yes | Proportion |
| employed_7days | work7days |  | work done in the last 7 days | 1 = yes | Proportion |
| mar_age | agemar |  | age when first marriage occurred | > 0 | Median |
| mar_witpartner | curmar |  | marital status | 1 = married | Proportion |
|  |  |  |  | 2 = living together |  |
| prghlthaccess | prgcare |  | health facility visit for antenatal care | 1 = yes | Proportion |
| pregnow_prop | pregnant |  | pregnancy status | 1 = yes | Proportion |
| prp3msick | sick3mo | Household Roster | Has been very sick for at least 3 months during the past 12 months | 1 = Yes | Proportion |
| prpelderly | agegroup5population |  | proportion of elderly population | integer | Proportion |
| prpyoung | agegroup5population |  | proportion youn (0 to 14 years) | integer | Proportion |

## Supplemental Table 6. Indicators for Tanzania

| **Variable Computed** | **Variable Used** | **Extracted From** | **Name** | **Code** | **Computation** |
| --- | --- | --- | --- | --- | --- |
| btwatersource | watersource | Household Interviews | source of water | 11= piped into dwelling | Proportion |
|  |  |  |  | 12 = piped into yard/plot |  |
|  |  |  |  | 13 = public tap/standpipe |  |
|  |  |  |  | 21 = tube well or borehole |  |
|  |  |  |  | 31 = protected well |  |
|  |  |  |  | 41 = protected spring |  |
|  |  |  |  | 91 = bottled water |  |
| bttoilettype | toilettype_tz |  | type of toilet facility | 12 = flush to septic tank | Proportion |
|  |  |  |  | 13 = flush to pit latrine |  |
|  |  |  |  | 16 = flush to piped sewer system |  |
|  |  |  |  | 22 = ventilated improved pit latrine (VIP) |  |
| ytoiletshare | toiletshare |  | shared toilet facility | 1 = yes | Proportion |
| yhaveelect | hhqitems_a |  | availability of electricity | 1 = yes | Proportion |
| yhaveradio | hhqitems_b |  | available working radio | 1 = yes | Proportion |
| yhavetele | hhqitems_c |  | available working television | 1 = yes | Proportion |
| yhavecell | hhqitems_d |  | available working mobile phone | 1 = yes | Proportion |
| yhaverefrig | hhqitems_e |  | available working refrigerator | 1 = yes | Proportion |
| btcookingfuel | cookingfuel |  | Type of cooking fuel | 1 = electricity | Proportion |
|  |  |  |  | 2 = lpg / natural gas |  |
|  |  |  |  | 3 = biogas |  |
|  |  |  |  | 4 = paraffin / kerosene |  |
| btmatfloor | matfloor |  | material of floor | 31 = parquet or polished wood | Proportion |
|  |  |  |  | 32 = vinyl tiles / vinyl carpet |  |
|  |  |  |  | 33 = ceramic tiles |  |
|  |  |  |  | 34 = cement |  |
|  |  |  |  | 35 = carpet |  |
| btmatroof | matroof |  | material of roof | 21 = corrugated iron | Proportion |
|  |  |  |  | 22 = tin cans |  |
|  |  |  |  | 31 = asbestos sheet / cement fiber |  |
|  |  |  |  | 32 = concrete |  |
|  |  |  |  | 33 = ceramic / clay tiles |  |
| btmatexwalls | matexwalls |  | material of exterior wall | 31 = cement | Proportion |
|  |  |  |  | 32 = stone with lime/ cement |  |
|  |  |  |  | 33 = bricks |  |
|  |  |  |  | 34 = cement blocks |  |
| roomsleepHH | roomsleep, rostercount |  | number of rooms for sleeping | integer | Median |
| yownmoto | hhqown_b |  | available working motor cycle / scooter | 1 = yes | Proportion |
| yowncar | hhqown_c |  | available working car | 1 = yes | Proportion |
| noecosrept | econsup12_a |  | economic support within 12 months | 1 = yes | Proportion |
| childHHratio | childcount, rostercount |  | number of children per household | integer | Median |
| womenprop | gender | Adult Interviews | proportion of women | 2 = female | Proportion |
| rel_literacy | schcom |  | highest level of school completed | 2 = lvl _1 (primary, 1-7) | Proportion |
|  |  |  |  | 3 = lvl_2 (secondary, 1-3 or A-B) |  |
|  |  |  |  | 4 = lvl_2 (high school, 4-5 or D-E) |  |
|  |  |  |  | 5 = lvl_3 (short cycle tertiary) |  |
|  |  |  |  | 6 = lvl_3 (bachelor's program) |  |
|  |  |  |  | 7 = lvl_3 (master's program) |  |
|  |  |  |  | 8 = lvl_3 (doctoral program) |  |
| employed_12mth | work12mo |  | work done in the last 12 months | 1 = yes | Proportion |
| employed_7days | work7days |  | work done in the last 7 days | 1 = yes | Proportion |
| mar_age | agemar |  | age when first marriage occurred | > 0 | Median |
| mar_witpartner | curmar |  | marital status | 1 = married | Proportion |
|  |  |  |  | 2 = living together |  |
| prghlthaccess | prgcare |  | health facility visit for antenatal care | 1 = yes | Proportion |
| prgnow_prop | pregnant |  | pregnancy status | 1 = yes | Proportion |
| prp3msick | sick3mo | Household Roster | Has been very sick for at least 3 months during the past 12 months | 1 = Yes | Proportion |
| prpelderly | Agegroup5population |  | Proportion of elderly | integer | Proportion |
| prpyoung | Agegroup5population |  | Proportion of young (0 to 14 years) | integer | Proportion |

## Supplemental Table 7. Indicators for Uganda

| **Variable Computed** | **Variable Used** | **Extracted From** | **Name** | **Code** | **Computation** |
| --- | --- | --- | --- | --- | --- |
| btwatersource | watersource | Household Interviews | source of water | 11= piped into dwelling | Proportion |
|  |  |  |  | 12 = piped into yard/plot |  |
|  |  |  |  | 13 = public tap/standpipe |  |
|  |  |  |  | 21 = tube well or borehole |  |
|  |  |  |  | 31 = protected well |  |
|  |  |  |  | 41 = protected spring |  |
|  |  |  |  | 91 = bottled water |  |
| bttoilettype | toilettype |  | type of toilet facility | 11 = flush or pour flush toilet | Proportion |
|  |  |  |  | 22 = ventilated improved pit laterine |  |
| ytoiletshare | toiletshare |  | shared toilet facility | 1 = yes | Proportion |
| yhaveelect | hhqitems_a |  | availability of electricity | 1 = yes | Proportion |
| yhaveradio | hhqitems_b |  | available working radio | 1 = yes | Proportion |
| yhavetele | hhqitems_c |  | available working television | 1 = yes | Proportion |
| yhavecell | hhqitems_d |  | available working mobile phone | 1 = yes | Proportion |
| yhaverefrig | hhqitems_e |  | available working refrigerator | 1 = yes | Proportion |
| btcookingfuel | cookingfuel |  | Type of cooking fuel | 1 = electricity | Proportion |
|  |  |  |  | 2 = lpg / natural gas |  |
|  |  |  |  | 3 = biogas |  |
|  |  |  |  | 4 = paraffin / kerosene |  |
| btmatfloor | matfloor |  | material of floor | 31 = parquet or polished wood | Proportion |
|  |  |  |  | 32 = vinyl tiles / vinyl carpet |  |
|  |  |  |  | 33 = ceramic tiles |  |
|  |  |  |  | 34 = cement |  |
|  |  |  |  | 35 = carpet |  |
| btmatroof | matroof |  | material of roof | 21 = corrugated iron | Proportion |
|  |  |  |  | 22 = tin cans |  |
|  |  |  |  | 31 = asbestos sheet / cement fiber |  |
|  |  |  |  | 32 = concrete |  |
|  |  |  |  | 33 = ceramic / clay tiles |  |
| btmatexwalls | matexwalls |  | material of exterior wall | 31 = cement | Proportion |
|  |  |  |  | 32 = stone with lime/ cement |  |
|  |  |  |  | 33 = bricks |  |
|  |  |  |  | 34 = cement blocks |  |
| roomsleepHH | roomsleep, rostercount |  | number of rooms for sleeping | integer | Median |
| yownmoto | hhqown_b |  | available working motor cycle / scooter | 1 = yes | Proportion |
| yowncar | hhqown_c |  | available working car | 1 = yes | Proportion |
| noecosrept | econsup12_a |  | economic support within 12 months | 1 = yes | Proportion |
| childHHratio | childcount, rostercount |  | number of children per household | integer | Median |
| HHSize | rostercount |  | size of household | integer | Median |
| womenprop | gender | Adult Interviews | proportion of women | 2 = women | Proportion |
| rel_literacy | schcom |  | highest level of school completed | 2 = lvl _1 (primary, 1-7) | Proportion |
|  |  |  |  | 3 = lvl_2 (secondary, 1-3 or A-B) |  |
|  |  |  |  | 4 = lvl_2 (high school, 4-5 or D-E) |  |
|  |  |  |  | 5 = lvl_3 (short cycle tertiary) |  |
|  |  |  |  | 6 = lvl_3 (bachelor's program) |  |
|  |  |  |  | 7 = lvl_3 (master's program) |  |
|  |  |  |  | 8 = lvl_3 (doctoral program) |  |
| employed_12mth | work12mo |  | work done in the last 12 months | 1 = yes | Proportion |
| employed_7days | work7days |  | work done in the last 7 days | 1 = yes | Proportion |
| mar_age | agemar |  | age when first marriage occurred | > 0 | Median |
| mar_witpartner | curmar |  | marital status | 1 = married | Proportion |
|  |  |  |  | 2 = living together |  |
| prghlthaccess | prgcare |  | health facility visit for antenatal care | 1 = yes | Proportion |
| prgnow_prop | pregnant |  | pregnancy status | 1 = yes | Proportion |
| prp3msick | sick3mo | Household Roster | Has been very sick for at least 3 months during the past 12 months | 1 = Yes | Proportion |
| prpelderly | agegroup5population |  | Proportion of elderly | integer | Proportion |
| prpyoung | agegroup5population |  | proportion of young (0 to 14 years) | integer | Proportion |

## Supplemental Table 8. Indicators for Zimbabwe

| **Variable Computed** | **Variable Used** | **Extracted From** | **Name** | **Code** | **Computation** |
| --- | --- | --- | --- | --- | --- |
| btwatersource | watersource | Household Interview | source of water | 11= piped into dwelling | Proportion |
|  |  |  |  | 12 = piped into yard/plot |  |
|  |  |  |  | 13 = public tap/standpipe |  |
|  |  |  |  | 21 = tube well or borehole |  |
|  |  |  |  | 31 = protected well |  |
|  |  |  |  | 41 = protected spring |  |
|  |  |  |  | 91 = bottled water |  |
| bttoilettype | toilettype |  | type of toilet facility | 11 = flush or pour flush toilet | Proportion |
|  |  |  |  | 22 = ventilated improved pit laterine |  |
| ytoiletshare | toiletshare |  | shared toilet facility | 1 = yes | Proportion |
| yhaveelect | hhqitems_a |  | availability of electricity | 1 = yes | Proportion |
| yhaveradio | hhqitems_b |  | available working radio | 1 = yes | Proportion |
| yhavetele | hhqitems_c |  | available working television | 1 = yes | Proportion |
| yhavecell | hhqitems_d |  | available working mobile phone | 1 = yes | Proportion |
| yhaverefrig | hhqitems_e |  | available working refrigerator | 1 = yes | Proportion |
| btcookingfuel | cookingfuel |  | Type of cookingfuel | 1 = electricity | Proportion |
|  |  |  |  | 2 = lpg / natural gas |  |
|  |  |  |  | 3 = biogas |  |
|  |  |  |  | 4 = paraffin / kerosene |  |
| btmatfloor | matfloor |  | material of floor | 31 = parquet or polished wood | Proportion |
|  |  |  |  | 32 = vinyl tiles / vinyl carpet |  |
|  |  |  |  | 33 = ceramic tiles |  |
|  |  |  |  | 34 = cement |  |
|  |  |  |  | 35 = carpet |  |
| btmatroof | matroof |  | material of roof | 21 = corrugated iron | Proportion |
|  |  |  |  | 22 = tin cans |  |
|  |  |  |  | 31 = asbestos sheet / cement fiber |  |
|  |  |  |  | 32 = concrete |  |
|  |  |  |  | 33 = ceramic / clay tiles |  |
| btmatexwalls | matexwalls |  | material of exterior wall | 31 = cement | Proportion |
|  |  |  |  | 32 = stone with lime/ cement |  |
|  |  |  |  | 33 = bricks |  |
|  |  |  |  | 34 = cement blocks |  |
| roomsleepHH | roomsleep, rostercount |  | number of rooms for sleeping | integer | Median |
| yownmoto | hhqown_b |  | available working motor cycle / scooter | 1 = yes | Proportion |
| yowncar | hhqown_c |  | available working car | 1 = yes | Proportion |
| noecosrept | econsup12_a_zw |  | economic support within 12 months | 1 = yes | Proportion |
| childHHratio | childcount/rostercount |  | number of children per household | integer | Median |
| HHSize | rostercount |  | size of household | integer | Median |
| womenprop | gender | Adult Interview | proportion of women | 2 = women | Proportion |
| rel_literacy | schcom |  | highest level of school completed | 2 = lvl _1 (primary, 1-7) | Proportion |
|  |  |  |  | 3 = lvl_2 (secondary, 1-3 or A-B) |  |
|  |  |  |  | 4 = lvl_2 (high school, 4-5 or D-E) |  |
|  |  |  |  | 5 = lvl_3 (short cycle tertiary) |  |
|  |  |  |  | 6 = lvl_3 (bachelor's program) |  |
|  |  |  |  | 7 = lvl_3 (master's program) |  |
|  |  |  |  | 8 = lvl_3 (doctoral program) |  |
| employed_12mth | work12mo |  | work done in the last 12 months | 1 = yes | Proportion |
| employed_7days | work7days |  | work done in the last 7 days | 1 = yes | Proportion |
| mar_age | agemar |  | age when first marriage occurred | > 0 | Median |
| mar_witpartner | curmar |  | marital status | 1 = married | Proportion |
|  |  |  |  | 2 = living together |  |
| prghlthaccess | prgcare |  | health facility visit for antenatal care | 1 = yes | Proportion |
| prgnow_prop | pregnant |  | pregnancy status | 1 = yes | Proportion |
| prp3msick | sick3mo | Household Roster | Has been very sick for at least 3 months during the past 12 months | 1 = Yes | Proportion |
| prpelderly | age |  | Age groups - 5-year groups | integer | Proportion |
| prpyoung | age |  | Age groups - 5-year groups | integer | Proportion |

## Supplemental Table 9. Indicators for Lesotho

| **Variable Computed** | **Variable Used** | **Extracted From** | **Name** | **Code** | **Computation** |
| --- | --- | --- | --- | --- | --- |
| btwatersource | watersource | Household Interviews | source of water | 11 = piped into dwelling | Proportion |
|  |  |  |  | 12 = piped into yard / plot |  |
|  |  |  |  | 13 = public tap / standpipe |  |
|  |  |  |  | 21 = tube well / borehole |  |
|  |  |  |  | 31 = protected well |  |
|  |  |  |  | 41 = protected spring |  |
|  |  |  |  | 91 = bottled water |  |
| bttoilettype | toilettype |  | type of toilet facility | 11 = flush or pour flush toilet | Proportion |
|  |  |  |  | 21 = traditional pit laterine |  |
|  |  |  |  | 22 = ventilated improved pit laterine (vip) |  |
| ytoiletshare | toiletshare |  | shared toilet facility | 1 = yes | Proportion |
| yhaveelect | hhqitems_a |  | availability of electricity | 1 = yes (A = electricity) | Proportion |
| yhaveradio | hhqitems_b |  | available working radio | 1 = yes ( B = A working radio) | Proportion |
| yhavetele | hhqitems_c |  | available working television | 1 = yes ( C = A working television) | Proportion |
| yhavecell | hhqitems_d |  | available working mobile phone | 1 = yes ( D = A working telephone) | Proportion |
| yhaverefrig | hhqitems_e |  | available working refrigerator | 1 = yes ( E = A working refrigerator | Proportion |
| btcookingfuel | cookingfuel |  | Type of cooking fuel | 1 = electricity | Proportion |
|  |  |  |  | 2 = lpg / natural gas |  |
|  |  |  |  | 3 = biogas |  |
|  |  |  |  | 4 = paraffin / kerosene |  |
| btmatfloor | matfloor |  | material of floor | 31 = parquet or polished wood | Proportion |
|  |  |  |  | 32 = vinyl tiles / vinyl carpet |  |
|  |  |  |  | 33 = ceramic tiles |  |
|  |  |  |  | 34 = cement |  |
|  |  |  |  | 35 = carpet |  |
| btmatroof | matroof |  | material of roof | 21 = corrugated iron | Proportion |
|  |  |  |  | 22 = tin cans |  |
|  |  |  |  | 31 = asbestos sheet / cement fiber |  |
|  |  |  |  | 32 = concrete |  |
|  |  |  |  | 33 = ceramic / clay tiles |  |
| btmatexwalls | matexwalls |  | material of exterior wall | 31 = cement | Proportion |
|  |  |  |  | 32 = stone with lime/ cement |  |
|  |  |  |  | 33 = bricks |  |
|  |  |  |  | 34 = cement blocks |  |
| roomsleepHH | roomsleep, rostercount |  | number of rooms for sleeping | integer | Median |
| yownmoto | hhqown_b |  | available working motor cycle / scooter | 1 = yes ( B = A working motor cycle / scoooter | Proportion |
| yowncar | hhqown_c |  | available working car | 1 = yes ( C = A working car or truck) | Proportion |
| fdinsecure4wk | hhqfood |  | Food security | 1 = yes ( no food in household) | Proportion |
| childHHratio | childcount, rostercount |  | number of children per house hold | integer | Median |
| HHSize | rostercount |  | size of house hold | integer | Median |
| womenprop | gender | Adult Interviews | proportion of women | 2 = female | Proportion |
| rel_literacy | schcom |  | highest level of school completed | 2 = lvl _1 (primary, 1-7) | Proportion |
|  |  |  |  | 3 = lvl_2 (secondary, 1-3 or A-B) |  |
|  |  |  |  | 4 = lvl_2 (high school, 4-5 or D-E) |  |
|  |  |  |  | 5 = lvl_3 (short cycle tertiary) |  |
|  |  |  |  | 6 = lvl_3 (bachelor's program) |  |
|  |  |  |  | 7 = lvl_3 (master's program) |  |
|  |  |  |  | 8 = lvl_3 (doctoral program) |  |
| employed_12mth | work12mo |  | work done in the last 12 months | 1 = yes | Proportion |
| employed_7days | work7days |  | work done in the last 7 days | 1 = yes | Proportion |
| mar_age | agemar |  | age when first marriage occurred | > 0 | Median |
| mar_witpartner | curmar |  | marital status | 1 = married | Proportion |
|  |  |  |  | 2 = living together |  |
| prghlthaccess | prgcare |  | health facility visit for antenatal care | 1 = yes | Proportion |
| pregnow_prop | pregnant |  | pregnancy status | 1 = yes | Proportion |
| prp3msick | sick3mo | Household Roster | Has been very sick for at least 3 months during the past 12 months | 1 = Yes | Proportion |
| prpelderly | age |  | Age groups - 5-year groups | integer | Proportion |
| prpyoung | age |  | Age groups - 5-year groups | integer | Proportion |

## Supplemental Table 10. Indicators for Eswatini

| **Variable Computed** | **Variable Used** | **Extracted From** | **Name** | **Code** | **Computation** |
| --- | --- | --- | --- | --- | --- |
| btwatersource | watersource | Household Interviews | source of water for household | 11 = piped into dwelling | Proportion |
|  |  |  |  | 12 =piped to yard/plots |  |
|  |  |  |  | 13 = public tap/standpipe |  |
|  |  |  |  | 21 = tube well or bore hole |  |
|  |  |  |  | 31 = protected well |  |
|  |  |  |  | 41 = protected spring |  |
|  |  |  |  | 91 = bottled water |  |
| bttoilettype | toilettype |  | type of toilet facility | 11 = flush or pour flush toilet | Proportion |
|  |  |  |  | 21 = traditional pit latrine |  |
|  |  |  |  | 22 = ventilated improved pit latrine |  |
| ytoiletshare | toiletshare |  | sharing toilet facility | 1 = yes | Proportion |
| yhaveelect | hhqitems_a |  | electricity in household | 1 = yes | Proportion |
| yhaveradio | hhqitems_b |  | radio in household | 1 = yes | Proportion |
| yhavetele | hhqitems_c |  | television in household | 1 = yes | Proportion |
| yhavecell | hhqitems_d |  | mobile phone in household | 1 = yes | Proportion |
| yhaverefrig | hhqitems_e |  | refrigerator in household | 1 = yes | Proportion |
| btcookingfuel | cookingfuel |  | type of cooking fuel | 1 = electricity | Proportion |
|  |  |  |  | 2 = lpg / natural gas |  |
|  |  |  |  | 3 = biogas |  |
|  |  |  |  | 4 = paraffin / kerosene |  |
| btmatfloor | matfloor |  | material of floor | 31 = parquet or polished wood | Proportion |
|  |  |  |  | 32 = vinyl or asphalt strip |  |
|  |  |  |  | 33 = ceramic tiles |  |
|  |  |  |  | 34 = cement or terazo |  |
|  |  |  |  | 35 = carpet |  |
| btmatroof | matroof |  | material of roof | 21 = corrugated iron | Proportion |
|  |  |  |  | 22 = tin cans |  |
|  |  |  |  | 31 = asbestos sheet |  |
|  |  |  |  | 32 = concrete |  |
|  |  |  |  | 33 = tiles |  |
| btmatexwalls | matexwalls |  | material of exterior walls | 31 = cement | Proportion |
|  |  |  |  | 32 = stone with lime / cement |  |
|  |  |  |  | 33 = bricks |  |
|  |  |  |  | 34 = cement blocks |  |
| roomsleepHH | roomsleep, rostercount |  | number of rooms for sleeping | integer | Median |
| yownmoto | hhqown_b_sz |  | owned means of mobility | 1 = yes | Proportion |
| yowncar | hhqown_c_sz |  | owned means of mobility | 1 = yes | Proportion |
| noecosrept | econsup12_a |  | economic support in 12 months | 1 = yes | Proportion |
| childHHratio | childcount, rostercount |  | number of children per household | integer | Median |
| HHSize | rostercount |  | size of household | integer | Median |
| womenprop | gender | Adult Interviews | proportion of women | 2 = female | Proportion |
| rel_literacy | schcom_sz |  | literacy proportion | 8 = Level 1 (Primary, Year 7) | Proportion |
|  |  |  |  | 9 = Level 2 (Secondary, Form 1) |  |
|  |  |  |  | 10 = Level 2 (Secondary, Form 2) |  |
|  |  |  |  | 11 = Level 2 (Secondary, Form 3) |  |
|  |  |  |  | 12 = Level 2 (Secondary, Form 4) |  |
|  |  |  |  | 13 = Level 2 (Secondary, Form 5) |  |
|  |  |  |  | 14 = Level 2 (Secondary, Form 6) |  |
|  |  |  |  | 15 = Level 3 (Short Cycle, Tertiary) |  |
|  |  |  |  | 16 = Level 3 (Bachelor's Program) |  |
|  |  |  |  | 17 = Level 3 (Master's Program) |  |
|  |  |  |  | 18 = Level 3 (Doctoral Program) |  |
| employed_12mth | work12mo |  | 12 months of employment | 1 = yes | Proportion |
| employed_7days | work7days |  | 7 days of employment | 1 = yes | Proportion |
| mar_age | agemar |  | age at which first marriage occurred | > 0 = ages greater than 0 for first time marriage | Median |
| mar_witpartner | curmar |  | married or living together | 1 = married | Proportion |
|  |  |  |  | 2 = living with partner |  |
| prghlthaccess | prgcare |  | visiting healthcare facility | 1 = yes | Proportion |
| pregnow_prop | pregnant |  | pregnant | 1 = yes | Proportion |
| prp3msick | sick3mo | Household Roster | Has been very sick for at least 3 months during the past 12 months | 1=Yes | Proportion |
| prpelderly | agegroup5population |  | proportion of elderly | integer | Proportion |
| prpyoung | agegroup5population |  | proportion of young (0 to 14 years) | integer | Proportion |

## Supplemental Table 11. Indicators for Nigeria

| **Variable Computed** | **Variable Used** | **Extracted From** | **Name** | **Code** | **Computation** |
| --- | --- | --- | --- | --- | --- |
| btwatersource | watersource | Household Interviews | source of water | 11= piped into dwelling | Proportion |
|  |  |  |  | 12 = piped into yard/plot |  |
|  |  |  |  | 13 = public tap/standpipe |  |
|  |  |  |  | 14 = piped to neighbor |  |
|  |  |  |  | 21 = tube well or borehole |  |
|  |  |  |  | 31 = protected well |  |
|  |  |  |  | 41 = protected spring |  |
|  |  |  |  | 51 = rainwater |  |
|  |  |  |  | 91 = bottled water |  |
|  |  |  |  | 92 = sachet (pure) water |  |
| bttoilettype | toilettype |  | type of toilet facility | 11 = flush to piped sewer system | Proportion |
|  |  |  |  | 12 = flush to septic tank |  |
|  |  |  |  | 13 = flush to pit latrine |  |
|  |  |  |  | 21 = ventilated improved pit latrine (VIP) |  |
|  |  |  |  | 22 = ventilated improved pit laterine |  |
|  |  |  |  | 31 = composting toilet |  |
| ytoiletshare | toiletshare |  | shared toilet facility | 1 = yes | Proportion |
| toiletloc_ng | toiletloc_ng |  | toilet location | 1 = in own dwelling | Proportion |
|  |  |  |  | 2 = In yard/plot |  |
| yhaveelect | haveelect |  | availability of electricity | 1 = yes | Proportion |
| yhaveradio | haveradio |  | available working radio | 1 = yes | Proportion |
| yhavetele | havetele |  | available working television | 1 = yes | Proportion |
| yhaverefrig | haverefrig |  | available working refrigerator | 1 = yes | Proportion |
| yhavecomp | havecomp |  | available working computer | 1 = yes | Proportion |
| yownbankacc | ownbankacc |  | functioning bank account | 1 = yes | Proportion |
| ysepkitchenroom_ng | sepkitchenroom_ng |  | separate room for kitchen | 1 = yes | Proportion |
| btcookingfuel | cookingfuel |  | type of cooking fuel | 1 = electricity | Proportion |
|  |  |  |  | 2 = liquid propane gas |  |
|  |  |  |  | 3 = natural gas |  |
|  |  |  |  | 4 = biogas |  |
|  |  |  |  | 5 = paraffin/kerosene |  |
| btmatfloor | matfloor |  | material of floor | 31 = parquet or polished wood | Proportion |
|  |  |  |  | 32 = vinyl tiles / vinyl carpet |  |
|  |  |  |  | 33 = ceramic tiles |  |
|  |  |  |  | 34 = cement |  |
|  |  |  |  | 35 = carpet |  |
|  |  |  |  | 36 = terazzo |  |
| btmatroof | matroof |  | material of roof | 32 = Metal | Proportion |
|  |  |  |  | 34 = Calamine/cement fiber |  |
|  |  |  |  | 35 = ceramic tiles |  |
|  |  |  |  | 36 = cement |  |
|  |  |  |  | 37 = roofing shingles |  |
| btmatexwalls | matexwalls |  | material of exterior wall | 32 = unbaked bricks covered | Proportion |
|  |  |  |  | 33 = with plaster |  |
|  |  |  |  | 34 = bricks |  |
|  |  |  |  | 35 = cement blocks |  |
|  |  |  |  | 36 = cement |  |
|  |  |  |  | 37 = stone with lime/cement |  |
| roomsleepHH | roomsleep |  | number of rooms for sleeping | integer | Median |
| yownmoto | ownmoto |  | available working motor cycle / scooter | 1 = yes | Proportion |
| yowncar | owncar |  | available working car | 1 = yes | Proportion |
| yownknapep_ng | ownknapep_ng |  | available working keke napep | 1 = yes | Proportion |
| childHHratio | childcount, rostercount |  | number of children per household | integer | Median |
| HHSize | rostercount |  | size of household | integer | Median |
| womenprop | gender | Household Interviews | proportion of women | 2 = female | Proportion |
| rel_literacy | schlhi |  | highest level of school completed | 1 = primary | Proportion |
|  |  |  |  | 2 = junior secondary |  |
|  |  |  |  | 3 = senior secondary |  |
|  |  |  |  | 4 = A level |  |
|  |  |  |  | 5 = university or above |  |
|  |  |  |  | 6 = techinical or vocational |  |
|  |  |  |  | 7 = adult literacy (no formal education) |  |
| employed_12mth | work12mo |  | work done in the last 12 months | 1 = yes | Proportion |
| employed_7days | work7days |  | work done in the last 7 days | 1 = yes | Proportion |
| mar_age | agemar |  | age when first marriage occurred | > 0 | Median |
| mar_witpartner | maritalstatus |  | marital status | 2 = living together | Proportion |
| prghlthaccess | prgcare |  | visit hostipal for antenatal care | 1 = yes | Median |
| pregnow_prop | pregnant |  | pregnant | 1 = yes | Proportion |

## Supplemental Table 12. Indicators for Botswana

| **Variable Computed** | **Variable Used** | **Extracted From** | **Name** | **Code** | **Computation** |
| --- | --- | --- | --- | --- | --- |
| btwatersource | watersource | Household Interview | source of water | 11= piped into dwelling | Proportion |
|  |  |  |  | 12 = piped into yard/plot |  |
|  |  |  |  | 13 = public tap/standpipe |  |
|  |  |  |  | 21 = tube well or borehole |  |
|  |  |  |  | 31 = protected well |  |
|  |  |  |  | 41 = protected spring |  |
|  |  |  |  | 91 = bottled water |  |
| bttoilettype | toilettype |  | type of toilet facility | 11 = flush or pour flush toilet | Proportion |
|  |  |  |  | 22 = ventilated improved pit latrine |  |
| ytoiletshare | toiletshare |  | shared toilet facility | 1 = yes | Proportion |
| yhaveelect | hhqitems_a |  | availability of electricity | 1 = yes | Proportion |
| yhaveradio | hhqitems_b |  | available working radio | 1 = yes | Proportion |
| yhavetele | hhqitems_c |  | available working television | 1 = yes | Proportion |
| yhavecell | hhqitems_d |  | available working mobile phone | 1 = yes | Proportion |
| yhaverefrig | hhqitems_e |  | available working refrigerator | 1 = yes | Proportion |
| yhaveintnet | hhqitems_f_bw |  | availability of internet | 1 = yes | Proportion |
| yhavecomp | hhqitems_g_bw |  | availability of computer | 1 = yes | Proportion |
| btcookingfuel | cookingfuel |  | Type of cooking fuel | 1 = electricity | Proportion |
|  |  |  |  | 2 = lpg / natural gas |  |
|  |  |  |  | 3 = Biogas |  |
| btmatfloor | matfloor |  | material of floor | 32 = Vinyl or Asphalt Strip | Proportion |
|  |  |  |  | 33 = ceramic tiles |  |
|  |  |  |  | 34 = cement |  |
|  |  |  |  | 35 = carpet |  |
| btmatroof | matroof |  | material of roof | 21 = corrugated iron | Proportion |
|  |  |  |  | 22 = Tin Cans |  |
|  |  |  |  | 31 = asbestos sheet / cement fiber |  |
|  |  |  |  | 32 = concrete |  |
|  |  |  |  | 33 = tiles |  |
| btmatexwalls | matexwalls |  | material of exterior wall | 31 = cement | Proportion |
|  |  |  |  | 32 = stone with lime/ cement |  |
|  |  |  |  | 33 = bricks |  |
|  |  |  |  | 34 = cement blocks |  |
| yownmoto | hhqown_b |  | available working motor cycle / scooter | 1 = yes | Proportion |
| yowncar | hhqown_c |  | available working car | 1 = yes | Proportion |
| noecosrept | econsup12_a |  | economic support within 12 months | 1 = yes | Proportion |
| womenprop | gender | Adult Individual Interview | proportion of women | 2 = gender | Proportion |
| rel_literacy | schcom |  | highest level of school completed | 0 = Primary | Proportion |
|  |  |  |  | 1 = Secondary |  |
|  |  |  |  | 2 = Higher |  |
| employed_12mth | work12mo |  | work done in the last 12 months | 1 = yes | Proportion |
| employed_7days | work7days |  | work done in the last 7 days | 1 = yes | Proportion |
| mar_age | agemar |  | age when first marriage occurred | > 0 | Median |
| mar_witpartner | curmar |  | marital status | 1 = married | Proportion |
|  |  |  |  | 2 = living together |  |
| prghlthaccess | prgcare |  | health facility visit for antenatal care | 1 = yes | Proportion |
| prgnow_prop | pregnant |  | pregnancy status | 1 = yes | Proportion |
| prp3msick | sick3mo | Household Roster | Has been very sick for at least 3 months during the past 12 months | 1 = Yes | Proportion |
| prpelderly | agegroup5population |  | proportion of elderly | integer | Proportion |
| prpyoung | agegroup5population |  | proportion of young (0 to 14 years) | integer | Proportion |

## Supplemental Table 13: Descriptive Statistics for the PSI across the selected countries

| Region | N | Minimum | Maximum | Mean | Median | Standard Deviation | Skewness |
| --- | --- | --- | --- | --- | --- | --- | --- |
| Botswana | 30 | 2.751 | 4.187 | 3.266 | 3.179 | 0.362 | 0.754 |
| Eswatini | 55 | 3.448 | 4.282 | 3.839 | 3.817 | 0.156 | 0.235 |
| Lesotho | 78 | 2.723 | 3.376 | 3.121 | 3.15 | 0.167 | -0.51 |
| Malawi | 252 | 3.076 | 5.04 | 3.815 | 3.755 | 0.394 | 1.024 |
| Mozambique | 129 | 4.485 | 5.057 | 4.754 | 4.746 | 0.133 | 0.119 |
| Nigeria | 775 | 2.819 | 4.217 | 3.454 | 3.376 | 0.303 | 0.501 |
| Tanzania | 184 | 2.786 | 4.173 | 3.443 | 3.418 | 0.232 | 0.575 |
| Uganda | 166 | 2.036 | 3.339 | 2.528 | 2.474 | 0.267 | 0.636 |
| Zambia | 115 | 2.363 | 4.759 | 3.805 | 3.927 | 0.556 | -0.774 |
| Zimbabwe | 91 | 2.589 | 3.727 | 3.293 | 3.354 | 0.218 | -0.567 |

N – Number of Spatial Units (LGA or 3rd Order Spatial Units)

## Supplemental Table 14: Breakpoints and Bounds of the PSI Classes across countries investigated

| Country | Class | lower_bound | upper_bound | breakpoint_value | No. Spatial Units |
| --- | --- | --- | --- | --- | --- |
| BAIS | Low | 2.751 | 2.892 | 2.751 | 30 |
| BAIS | Relatively Low | 2.892 | 3.308 | 2.892 | 30 |
| BAIS | Relatively Moderate | 3.308 | 3.588 | 3.308 | 30 |
| BAIS | Relatively High | 3.588 | 3.937 | 3.588 | 30 |
| BAIS | Very High | 3.937 | 4.187 | 3.937 | 30 |
| ESWA | Low | 3.448 | 3.526 | 3.448 | 55 |
| ESWA | Relatively Low | 3.526 | 3.800 | 3.526 | 55 |
| ESWA | Relatively Moderate | 3.800 | 3.901 | 3.800 | 55 |
| ESWA | Relatively High | 3.901 | 4.057 | 3.901 | 55 |
| ESWA | Very High | 4.057 | 4.282 | 4.057 | 55 |
| LE | Low | 2.723 | 2.896 | 2.723 | 78 |
| LE | Relatively Low | 2.896 | 3.039 | 2.896 | 78 |
| LE | Relatively Moderate | 3.039 | 3.140 | 3.039 | 78 |
| LE | Relatively High | 3.140 | 3.243 | 3.140 | 78 |
| LE | Very High | 3.243 | 3.376 | 3.243 | 78 |
| MW | Low | 3.076 | 3.508 | 3.076 | 252 |
| MW | Relatively Low | 3.508 | 3.775 | 3.508 | 252 |
| MW | Relatively Moderate | 3.775 | 4.097 | 3.775 | 252 |
| MW | Relatively High | 4.097 | 4.546 | 4.097 | 252 |
| MW | Very High | 4.546 | 5.040 | 4.546 | 252 |
| MZ | Low | 4.485 | 4.620 | 4.485 | 129 |
| MZ | Relatively Low | 4.620 | 4.711 | 4.620 | 129 |
| MZ | Relatively Moderate | 4.711 | 4.792 | 4.711 | 129 |
| MZ | Relatively High | 4.792 | 4.879 | 4.792 | 129 |
| MZ | Very High | 4.879 | 5.057 | 4.879 | 129 |
| NGA | Low | 2.819 | 3.156 | 2.819 | 775 |
| NGA | Relatively Low | 3.156 | 3.365 | 3.156 | 775 |
| NGA | Relatively Moderate | 3.365 | 3.624 | 3.365 | 775 |
| NGA | Relatively High | 3.624 | 3.891 | 3.624 | 775 |
| NGA | Very High | 3.891 | 4.217 | 3.891 | 775 |
| TZ | Low | 2.786 | 3.255 | 2.786 | 184 |
| TZ | Relatively Low | 3.255 | 3.412 | 3.255 | 184 |
| TZ | Relatively Moderate | 3.412 | 3.579 | 3.412 | 184 |
| TZ | Relatively High | 3.579 | 3.820 | 3.579 | 184 |
| TZ | Very High | 3.820 | 4.173 | 3.820 | 184 |
| UG | Low | 2.036 | 2.294 | 2.036 | 166 |
| UG | Relatively Low | 2.294 | 2.447 | 2.294 | 166 |
| UG | Relatively Moderate | 2.447 | 2.617 | 2.447 | 166 |
| UG | Relatively High | 2.617 | 2.850 | 2.617 | 166 |
| UG | Very High | 2.850 | 3.339 | 2.850 | 166 |
| ZAM | Low | 2.363 | 2.924 | 2.363 | 115 |
| ZAM | Relatively Low | 2.924372 | 3.437437 | 2.924372 | 115 |
| ZAM | Relatively Moderate | 3.437437 | 3.818052 | 3.437437 | 115 |
| ZAM | Relatively High | 3.818052 | 4.164809 | 3.818052 | 115 |
| ZAM | Very High | 4.164809 | 4.758544 | 4.164809 | 115 |
| ZIM | Low | 2.588877 | 2.741582 | 2.588877 | 91 |
| ZIM | Relatively Low | 2.741582 | 3.087514 | 2.741582 | 91 |
| ZIM | Relatively Moderate | 3.087514 | 3.269209 | 3.087514 | 91 |
| ZIM | Relatively High | 3.269209 | 3.47638 | 3.269209 | 91 |
| ZIM | Very High | 3.47638 | 3.726873 | 3.47638 | 91 |
