# Supplementary material for: Place susceptibility index mapping at local government scale from population-based survey for Sub-Saharan Africa
Source: PLoS One. 2026 Feb 17;21(2):e0340782. doi: 10.1371/journal.pone.0340782 (PMC12912589; doi:10.1371/journal.pone.0340782)
Supplement: Attachment — Submitted filename: Clara Oguji Comments and Suggestion.docx [file pone.0340782.s002.docx]

**Clara Oguji Comments and Suggestion**

- **Clarification of Misalignments in Correlations**

Section: Results, PSI Misaligned with Health and Life Expectancy

Issue: The manuscript mentions that countries like Eswatini, Lesotho, and Uganda show positive correlations with health and life expectancy, which contrasts with the expected negative correlation.

Revision: Add a sentence or paragraph explaining why these positive correlations might have occurred. Could there be unique contextual factors in these countries that skew the expected outcome? For example, do these countries have better-than-expected healthcare access or infrastructure that could influence the relationship?

- **Simplification of Statistical Terminology**

Section: Methods, Geostatistical Modeling

Issue: Terms like Deviance Information Criterion (DIC) and Watanabe-Akaike Information Criterion (WAIC) might be unclear to a broader audience.

Revision:Simplify or briefly explain the terms in the text or add a footnote.

- **Improvement of Figure and Table Labels**

Section: Results, PSI Distribution and PSI Classification Distribution Across Countries

Issue: The figure captions could be more descriptive to help readers interpret the data.

Revision: Add annotations or clarifying notes to the figures where necessary. For example, in Figure 2, explain briefly why certain countries have higher proportions in the "Very High" PSI class.

- **Streamline the Abstract**

**Section**: Abstract

Issue: The abstract is slightly long and contains some redundancy.

Revision: Trim some details while keeping key findings intact. "The Place Susceptibility Index (PSI) has the potential to be a critical tool for Ministries of Health and Public Health and disaster management organizations to prepare for and manage infectious disease outbreaks or natural disasters in Africa." Revised: "The Place Susceptibility Index (PSI) is a critical tool for preparing for infectious disease outbreaks and disasters in Africa.". Simplify technical descriptions in the abstract, e.g., "Jenk’s natural grouping algorithm" could be simplified to "a classification method."

**Section**: Results, **PSI Distribution**

Issue: The results section dives into statistical details immediately after the introductory sentence.

Revision:Start with a summary of key findings in simpler terms before diving into statistical specifics.

Example: "The PSI varied significantly across the 10 countries, with countries like Botswana showing lower susceptibility, while Zambia exhibited higher levels of susceptibility. This variation highlights the need for region-specific interventions." Then, proceed with the statistical details (e.g., means, medians).

- **Minor Typographical Corrections**

Section: Throughout the manuscript

Issue: Some inconsistency in the use of abbreviations and formatting (e.g., PSI vs. PSS, LGA vs. local government areas).

Revision: Ensure consistency with the use of abbreviations. For example, PSI should always be used the same way, and LGA should be expanded at first use and followed consistently (e.g., "Local Government Area (LGA)").

- **Enhanced Discussion on Policy Implications**

Section: Discussion, PSI and Public Health Interventions

Issue: The manuscript discusses PSI’s usefulness but lacks specific policy recommendations.

Revision: Expand the discussion by providing clear recommendations for action. Example: "Governments could use PSI to prioritize health interventions, focusing on regions with high susceptibility to outbreaks. This could include targeted vaccination campaigns or improvements in healthcare infrastructure."

- **Reference:** It’s important to make sure that the references in the manuscript, especially those about statistical methods and geospatial analysis, are current and still relevant. For example, the manuscript mentions using **Bayesian modeling** and other statistical methods. It’s a good idea to check that the studies cited for these methods are up-to-date, as newer research may offer better techniques or improved understanding of these methods. The manuscript also mentions using **geospatial tools** like **Enhanced Vegetation Index (EVI)** and **Land Surface Temperature (LST)**. These tools are constantly improving, so it’s important to verify that the sources cited are the most recent ones, as new technologies or research might offer better ways to analyze this data. The study uses **Population-based HIV Impact Assessments (PHIA)** data, which is often updated. If the manuscript cites older versions of this data, it would be helpful to update the reference to the most recent version, so the analysis is based on the latest available information. Additionally, it may be useful to check if any recent **review articles** or **meta-analyses** are available. These sources can summarize the latest research and give a broader view of the topic, which might strengthen the manuscript’s arguments. Updating the references ensures the manuscript is based on the latest research and is more credible.
