# Supplementary material for: Place susceptibility index mapping at local government scale from population-based survey for Sub-Saharan Africa
Source: PLoS One. 2026 Feb 17;21(2):e0340782. doi: 10.1371/journal.pone.0340782 (PMC12912589; doi:10.1371/journal.pone.0340782)
Supplement: Attachment — Submitted filename: Review_PONE-D-25-28111.docx [file pone.0340782.s003.docx]

**Comments**
The manuscript is interesting and focuses on developing high-resolution PSIs for selected African countries. However, some revisions are necessary to strengthen the work. I recommend the authors consider the following major revisions and clarifications:

**-**Include more appropriate and precise keywords.

**-** Incorporate recent studies on PSI, especially those linking urban resilience with rapid urbanization, and clearly highlight the research gap connected to the novelty of this work. The introduction should explicitly present the key research questions, critical knowledge gaps, and how this study addresses them, supported with relevant references.

**-** The paper does not clearly explain how sampling strategies were employed to address variability. Provide more detail on data preprocessing steps (e.g., handling outliers, normalization, and sampling strategy).

**-** Expand on the processing details, including data sources, category types, spatial resolution, cloud filtering methods, calculations performed, and software used.

**-** Discuss the advantages of applying the Bayesian spatial statistical model prior to its use in this study. The authors could consider applying feature sensitivity or feature selection techniques to enhance the model’s application and improve the reliability of the findings.

**-** Justify the rationale for selecting the Jenks Classification method.

**-** Address the uncertainty of PSI model outputs. To improve robustness, incorporate uncertainty analyses such as standard deviation, coefficient of variation, Monte Carlo simulations, or entropy measures for each crop type, enabling spatial quantification of prediction uncertainty.

**-** Provide policy recommendations for areas classified as high and low Place Susceptibility zones, linking them to strategies for improving urban sustainability indicators. Provide an analysis of the relationship between high purchasing power parity and the life expectancy and birth rate across the selected provinces, and explain how these relate to the PSI outcomes.

**-** Add a dedicated subsection. While some limitations are noted, it would be valuable to include a summary table highlighting current gaps and possible future solutions.

**-** Revise the conclusion to reconstruct the key findings and emphasize how the outputs can be practically applied by government agencies, policymakers, and urban planners.
